# Difelikefalin in the treatment of hemodialysis patients with pruritus: a systematic review and meta-analysis

**DOI:** 10.3389/fphar.2024.1476587

**Published:** 2024-12-06

**Authors:** Xiaoyue Cai, Guiming Wu, Yan Lin, Lichuan Yang

**Affiliations:** ^1^ Department of Nephrology, Chengdu BOE Hospital, Chengdu, China; ^2^ Department of Nephrology, Dazhou Central Hospital, Dazhou, China; ^3^ Department of Neurology, The First People’s Hospital of Shuangliu District, Chengdu, China; ^4^ Department of Nephrology, West China Hospital of Sichuan University, Chengdu, China

**Keywords:** difelikefalin, hemodialysis, pruritus, systematic review, meta-analysis

## Abstract

**Background and Objective:**

Uremic pruritus is a persistent condition that is difficult to cure in patients with end-stage renal disease who are having regular dialysis. It is highly prevalent, and current therapies have limited effectiveness and can cause significant adverse effects. Several trials have provided evidence that difelikefalin can be an effective treatment for uremic pruritus, with few side responses. However, it is important to note that the available evidence is limited. This study collected published randomized controlled trials for systematic review and Meta-analysis, to explore the efficacy and safety of difelikefalin treating uremic pruritus and to provide evidence-based medical evidence for clinical treatment.

**Methods:**

A systematic literature search was conducted in PubMed, EMBASE, Web of Science, the Cochrane Library Data from building libraries to 6 January 2024. We extracted data from eligible studies to analyze the efficacy and safety of difelikefalin in the treatment of hemodialysis patients with pruritus.

**Results:**

This study comprised 9 trials with 4,118 people. The meta-analysis demonstrated that difelikefalin is more effective than placebo in treating uremic pruritus. Specifically, difelikefalin resulted in a greater improvement in WI-NRS scores of at least 3 points from baseline (OR = 1.98) and at least 4 points from baseline (OR = 1.94). Additionally, difelikefalin led to a decrease in the total score of the 5-D itch scale (MD = 1.56), a decrease in the skindex-10 scale score (MD = 4.92), and a decrease in the WI-NRS scale score (MD = 0.91).

**Conclusion:**

Difelikefalin demonstrates significant efficacy in alleviating pruritus in individuals suffering from uremia. Althogh it has adverse events, they are mild.

## Introduction

Chronic kidney disease–associated pruritus (CKD-aP) is also known as uremic pruritus. Uremic pruritus is an intractable symptom in patients with end-stage kidney disease (ESKD) undergoing maintenance dialysis (Mettang and Kremer, 2015; Narita et al., 2022). Uremic pruritus is defined as ESKD people have itching that lasts for at least 3 months (Satti et al., 2019). It is a common, distressing, and underrecognized condition that affects more than 60% of patients undergoing hemodialysis, with 20%–40% of patients reporting moderate-to-severe pruritus (Aresi et al., 2019; Fishbane et al., 2020a). Persistent pruritus negatively affects physical and mental health. Uremic pruritus has also been associated with an increase in missed dialysis sessions, a higher risk of hospitalization, and an increase in mortality, particularly cardiovascular and infection-related mortality (Sukul et al., 2021).

The current management of uremic pruritus includes adequate dialysis, control of blood phosphorus, use of emollients, topical corticosteroids, immunosuppressants, antihistamines, gabapentin, pregabalin and Chinese medicine (Eusebio-Alpapara et al., 2020; Hercz et al., 2020). Despite the acknowledged importance of uremic pruritus to patients, with the exception of gabapentin, current evidence for its treatment is weak (Simonsen et al., 2017). However, it may have side effects. Natural or medicated topical treatments such as baby oil and moisturizers may cause burning or irritation in some patients (Lu et al., 2021). Topical corticosteroids or immunosuppressants may cause thin skin and decrease local resistance, thereby increasing the risk of infection. Tacrolimus is a calcineurin inhibitor and suppresses the production of IL-2 and has been demonstrated to be beneficial for uremic pruritus (Kuypers et al., 2004). However, topical tacrolimus carries a black-box warning of increased risk of skin cancer. Antihistamines are the most common clinical treatment for pruritus. Fifty-seven percent of doctors prescribed antihistamines as the first-line treatment for itch (Rayner et al., 2017). However, the use of antihistamines raises safety issues, especially in the elderly (Verduzco and Shirazian, 2020). The neuropathic/anticonvulsant agents Gabapentin and Pregabalin are the mostly widely studied systemic medications for the treatment of uremic pruritus. Their mechanism of action likely involves negative modulation of the alpha 2 delta subunit of voltage-gated calcium channels and/or inhibition of the release of calcitonin gene–related peptide (a mediator of itch) from primary afferent neurons. However, side effects such as somnolence and unsteadiness due to mononucleosis have been reported (Martin et al., 2020). Chinese medicine is also a means of uremic pruritus treatment, but at present there is little evidence of acupuncture therapy and Chinese herbal bath therapy (Lu et al., 2022).

Difelikefalin is a novel, selective kappa opioid receptor (KOR) agonist that does not readily enter the CNS owing to its hydrophilic D-amino acid peptidic structure (Viscusi et al., 2021). It exerts antipruritic effects by activating kappa opioid receptors in peripheral neurons and immune cells (Menzaghi et al., 2015). In phase 3 KALM-1and KALM-2 studies of intravenous (IV) difelikefalin in hemodialysis participants with moderate-to-severe pruritus, difelikefalin demonstrated significant reductions in itch intensity compared to placebo at week 12 (Fishbane et al., 2020b; Wooldridge et al., 2020). Some studies suggest that difelikefalin can effectively treat uremic pruritus with mild adverse reactions, but the evidence is limited. This study collected data from published randomized controlled trials for a systematic review and meta-analysis to explore the efficacy and safety of difelikefalin in the treatment of uremic pruritus and to provide evidence-based medical evidence for clinical treatment.

## Methods

### Inclusion and exclusion criteria

Make inclusion and exclusion criteria according to PICOS principles.

Inclusion criteria:

Population = patients age ≥18 years old with end-stage kidney disease who had been undergoing hemodialysis and who had moderate-to-severe pruritus.

Intervention = difelikefalin was used as an intervention.

Comparison = placebo was used as an intervention.

Outcomes = improvement of itching and the occurrence of adverse reactions.

Study Design = The study types were randomized controlled trials.

Exclusion criteria: Studies were excluded if the patients having pruritus not associated with chronic kidney disease; patients with chronic kidney disease who have not entered the hemodialysis stage; no control study; studies with unclear diagnostic criteria.

### Search strategy

A systematic literature search was conducted in PubMed, EMBASE, Web of Science and Cochrane Data from building libraries to 6 January 2024. The following Medical Subject Heading terms and free words were used, as shown in Table 1: “difelikefalin” or “CR854” and “pruritus” or“itch” and “chronic kidney disease” or “hemodialysis” or “uremia”.

**TABLE 1:** Search strategy.

Databases	PubMed, EMBASE, web of science, cochrane
Data	building libraries to 6 January 2024
#1	“difelikefalin”or “CR854”
#2	“pruritus” or “itch”
#3	“Chronic kidney disease” or “hemodialysis” or “uremia”
Search	#1 and #2 and #3

### Study selection and data collection

Two investigators independently screened the literature to identify studies that met inclusion criteria. Any discrepancies between the reviewers were resolved through discussion with a third reviewer. After reading the title and abstract to exclude obviously irrelevant literature, further reading the full text to determine inclusion. Including those reporting the use of difelikefalin in treating pruritus in hemodialysis patients. The reference lists of all identified studies were also examined to find additional eligible studies. Data was collected and entered into a spreadsheet. The extracted variables included author, study period, location, patient age, sex, clinical characteristics, treatment effect, and adverse reactions. Two investigators independently evaluated the risk of bias in the included studies and cross-checked the results. Risk of bias assessment was performed using the tool recommended by the Cochrane Assistance Network (Cai et al., 2021).

### Statistical analysis

The meta-analysis was conducted using RevMan 5.4. For dichotomous data, the Mantel-Haenszel method was employed, while the Inverse Variance method was used for continuous data. Mean differences (MDs) and 95% confidence intervals (CIs) were calculated for continuous data, and odds ratios (ORs) with 95% CIs were calculated for dichotomous data. The I^2^ statistic and Q test were used to evaluate statistical heterogeneity. I^2^ ≤ 50%, *P* > 0.05 indicated low heterogeneity, while higher values suggested substantial heterogeneity. Potential study bias was assessed using funnel plots.

## Results

### Search results and characteristics of the included studies

**FIGURE 1:**
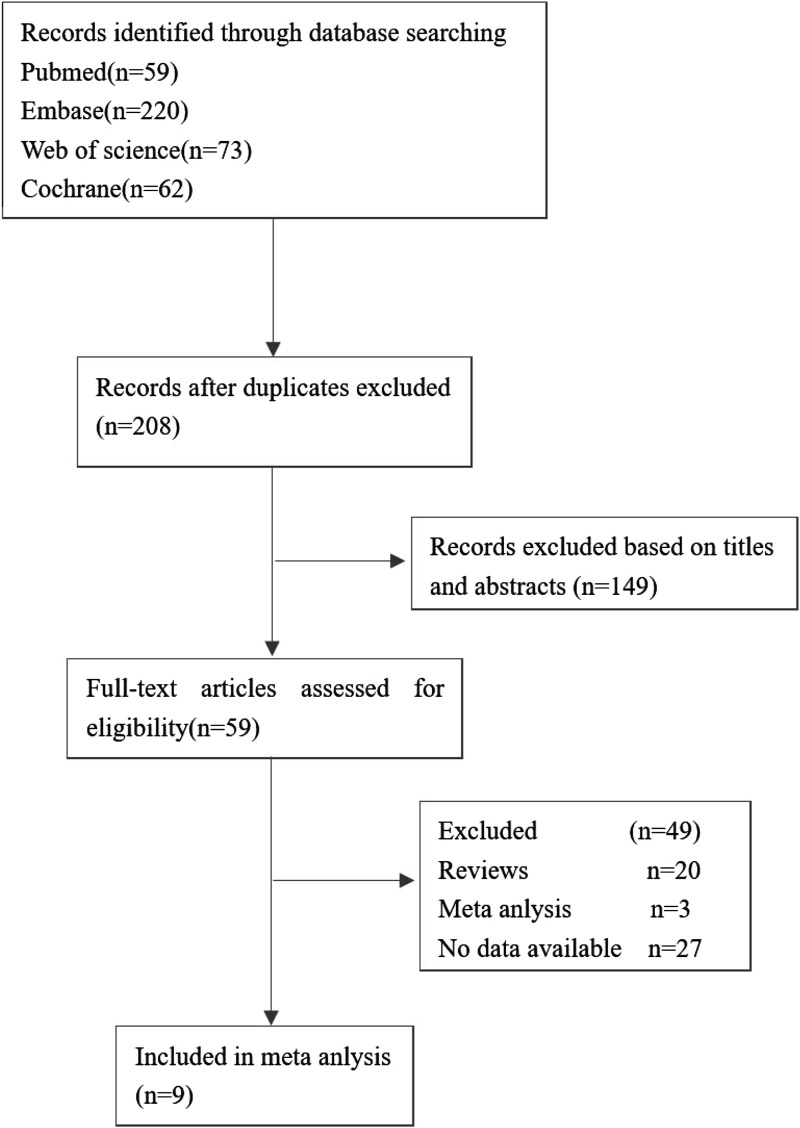
Flow diagram for selection of studies.

The flow of studies through the analysis is presented in Figure 1. 9 eligible studies involving 4,118 patients were enrolled in our study. The characteristics of the included studies are described in Table 2.

**TABLE 2:** Characteristics of the 9 studies included in the meta-analysis.

References	Country	Number of participants		Male (%)		Age, years		Time since initiation of hemodialysis, years		Type of study	Phase
		T1	T2	T1	T2	T1	T2	T1	T2		
Fishbane et al. (2020a)	United States	158	165	112 (59.3)	118 (62.8)	58.2 ± 11.2	56.8 ± 13.9	4.4 ± 4.0	4.7 ± 4.2	randomized, double-blind, placebo-controlled	3
Topf et al. (2022)	US, Europe, and Asia	426	425	246 (57.7)	258 (60.7)	59.1 ± 12.4	58.3 ± 13.5	3.9 ± 5.0	3.5 ± 4.8	randomized, placebo-controlled	3
Narita et al. (2022)	Japan	61	63	50 (82.0)	43 (70.5)	64.2 ± 11.2	64.1 ± 12.7	7.0 ± 6.5	6.8 ± 6.1	multicenter, randomized,double-blind,placebo-controlled	2
Narita et al. (2022)	Japan	61	63	45 (73.8)	43 (70.5)	65.6 ± 11.4	64.1 ± 12.7	6.7 ± 7.2	6.8 ± 6.1	multicenter, randomized,double-blind,placebo-controlled	2
Narita et al. (2022)	Japan	61	63	47 (77.0)	43 (70.5)	64.4 ± 11.7	64.1 ± 12.7	7.7 ± 6.5	6.8 ± 6.1	multicenter, randomized,double-blind,placebo-controlled	2
Yosipovitch et al. (2023)	United States	69	67	34 (49.3)	37 (55.2)	65.7 ± 11	65.6 ± 12.1	—	—	multicenter, randomized,double-blind,placebo-controlled	2
Yosipovitch et al. (2023)	United States	66	67	33 (50)	37 (55.2)	69 ± 12	65.6 ± 12.1	—	—	multicenter, randomized,double-blind,placebo-controlled	2
Yosipovitch et al. (2023)	United States	67	67	35 (52.2)	37 (55.2)	67.5 ± 10.7	65.6 ± 12.1	—	—	multicenter, randomized,double-blind,placebo-controlled	2
Weiner et al. (2023)	US, Europe, and Asia	426	425	249 (58.5)	258 (60.7)	59.1 ± 12.4	58.3 ± 13.5	3.9 ± 5.0	3.5 ± 4.8	multicenter, randomized, placebo-controlled	3
Spencer et al. (2023)	United States	16	14	9 (56.3)	7 (50)	58 ± 12.3	56 ± 9.5	—	—	randomized, double-blind, placebo-controlled	—
Wooldridge et al. (2020)	US, Europe, and Asia	235	236	135 (57.4)	139 (58.9)	59.7 ± 13.1	59.6 ± 13.1	4.8 ± 4.6	5.1 ± 4.3	Multicenter, multinational, double-blind, placebo-controlled	3
Fishbane et al. (2020b)	United States	44	45	26 (59.1)	28 (62.2)	57 ± 12.8	60 ± 14.3	5.4 ± 4.9	5.9 ± 4.9	randomized, double-blind, placebo-controlled	2
Fishbane et al. (2020b)	United States	41	45	23 (56.1)	28 (62.2)	59 ± 14.5	60 ± 14.3	6.3 ± 4.7	5.9 ± 4.9	randomized, double-blind, placebo-controlled	2
Fishbane et al. (2020b)	United States	44	45	28 (63.6)	28 (62.2)	56.5 ± 11.3	60 ± 14.3	5.5 ± 4.4	5.9 ± 4.9	randomized, double-blind, placebo-controlled	2
Fishbane et al. (2022)	US, Europe, and Asia	424	424	247 (58.3)	257 (60.6)	59 ± 12.3	58.4 ± 13.5	3.5 ± 3.6	3.9 ± 3.7	randomized, double-blind, placebo-controlled	3

T1: difelikefalin group; T2: placebo group.

### Results of bias risk assessment for included studies

**FIGURE 2:**
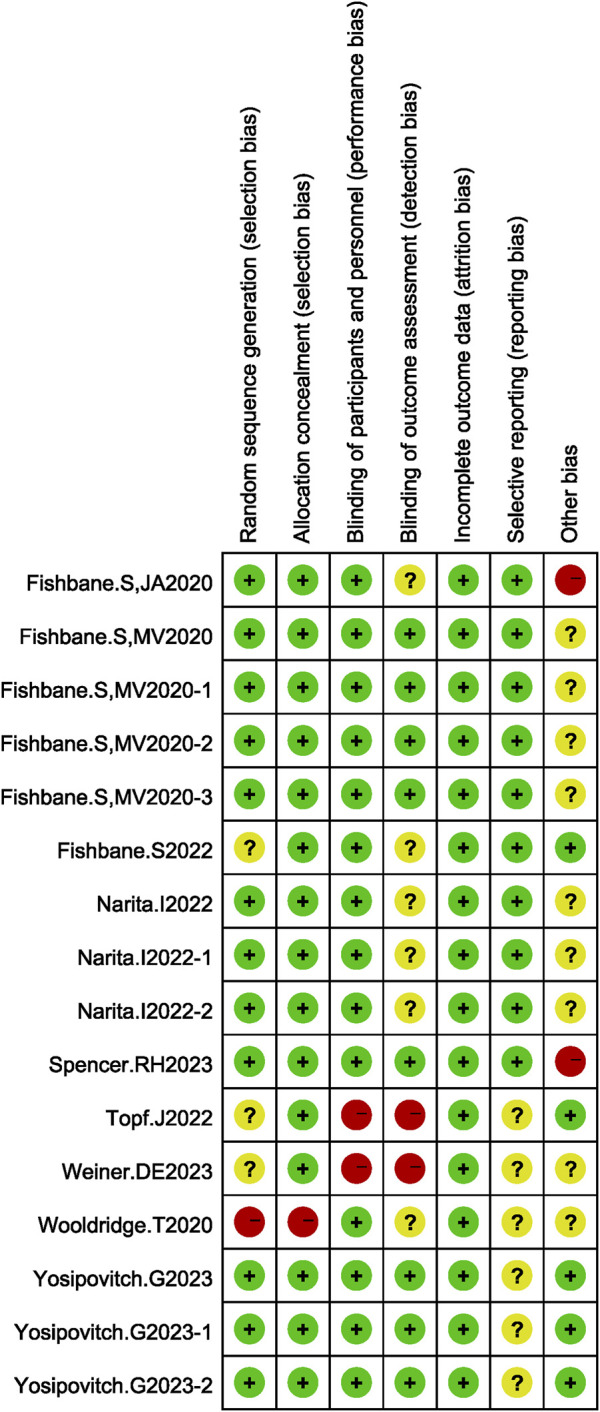
Results of bias risk assessment for included studies.

Among the studies included in this meta-analysis, there were 16 experiments in 9 studies most experiments with high quality and only minimal risk of bias. Among which 8 experiments scored 6points, only 2 experiments scored 2points (Figure 2).

### Analysis of outcomes

#### Score of WI-NRS improvement ≥3-Point

**FIGURE 3:**
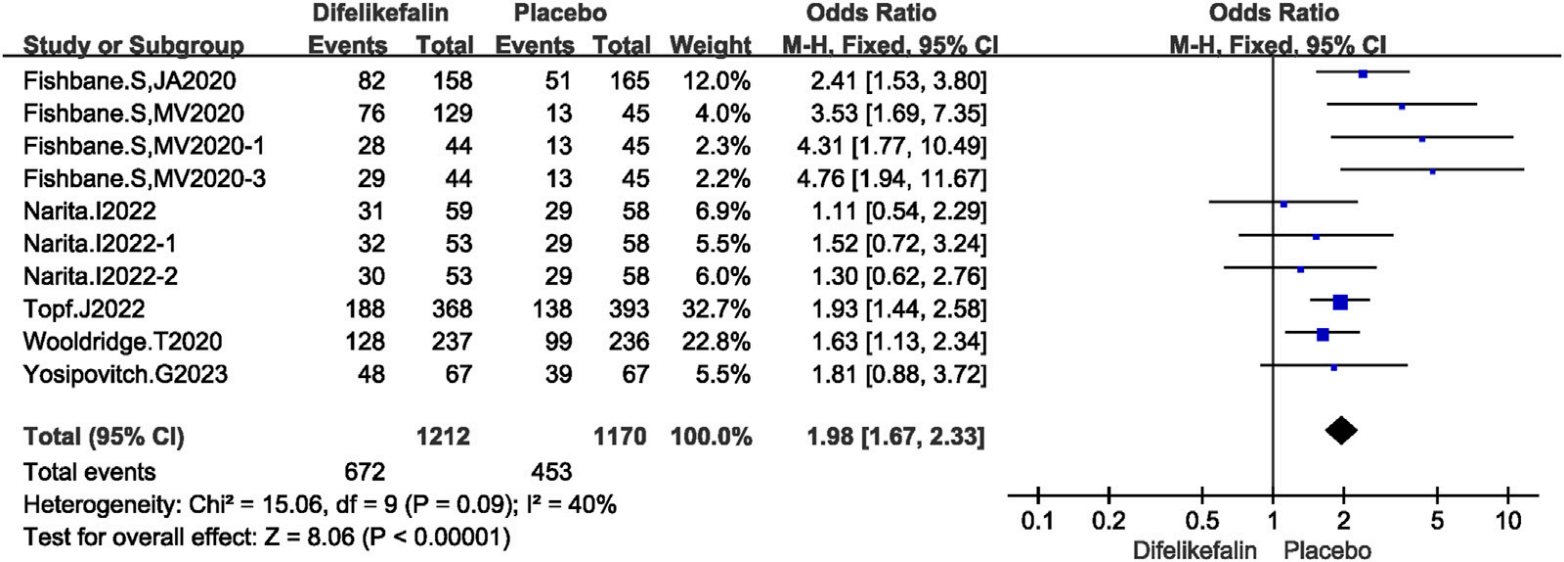
Forest plot showing the score of WI-NRS improvement ≥3-Point.

Six research, comprising 10 experiments, examined the improvement in WI-NRS scores of at least three points from the baseline. The I^2^ test revealed a value of 40%, which is less than the threshold of 50%, showing the presence of mild heterogeneity among the studies. Similarly, the Q test demonstrated a value of 0.09, which is greater than the threshold of 0.05, further confirming the presence of slight heterogeneity. The data was pooled using the fixed-effects model, resulting in an odds ratio (OR) of 1.98 (95% confidence interval [CI] 1.67–2.33, Z = 8.06, *P* < 0.00001) (Figure 3).

#### Score of WI-NRS improvement ≥4-Point

**FIGURE 4:**
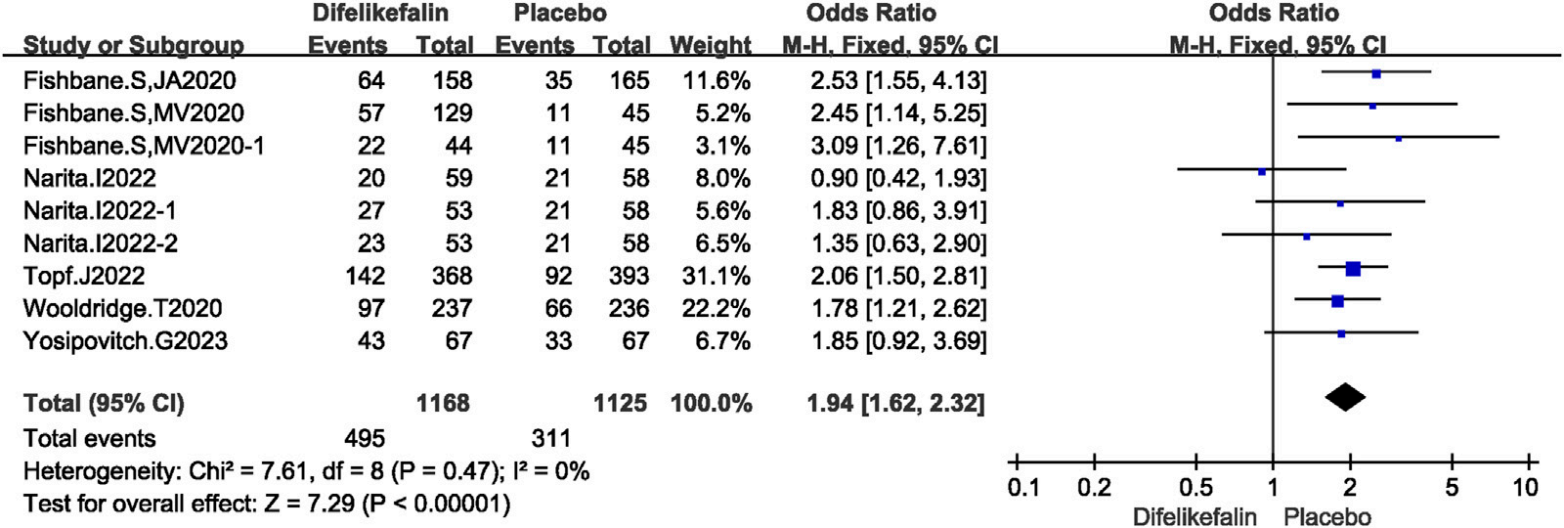
Forest plot showing the score of WI-NRS improvement ≥4-Point.

Six studies including nine experiments analyzed the score of WI-NRS improvement ≥4-point from baseline, The I^2^ test showed I^2^ = 0% < 50%, and Q test showed *P* = 0.47 > 0.05, indicating that no heterogeneity existed among the studies. The fixed-effects model was used to pool the data, yielding an OR of 1.94 (95% CI 1.62–2.32, Z = 7.29, *P* < 0.00001) (Figure 4).

#### 5-D itch scale total score decreases

**FIGURE 5:**
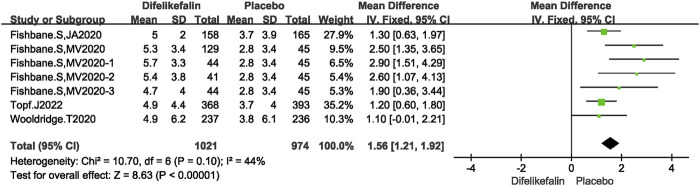
Forest plot showing the 5-D inch scale total score decrease.

Four research, consisting of seven experiments, examined the decrease in total scores on the 5-D itch scale. The I^2^ test revealed an I^2^ value of 44% which is less than 50%, and the Q test indicated a *P*-value of 0.1 which is greater than 0.05. This suggests that there was a modest level of heterogeneity among the studies. The data was pooled using the fixed-effects model, resulting in a mean difference (MD) of 1.56 (95% confidence interval [CI] 1.21–1.92, Z = 8.63, *P* < 0.00001) (Figure 5).

#### Skindex-10 scale score decrease

**FIGURE 6:**
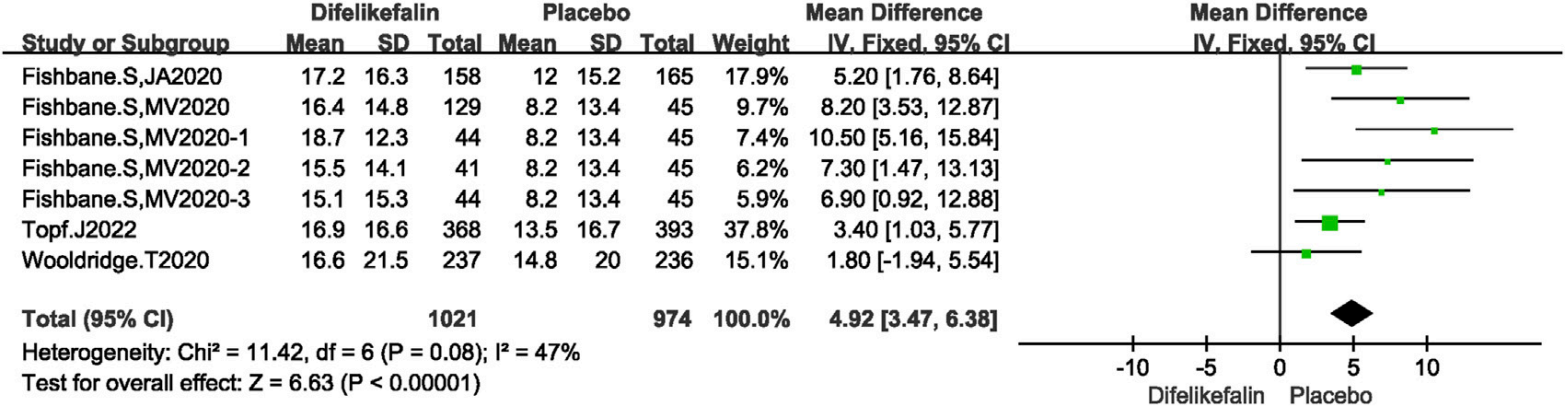
Forest plot showing the Skindex-10 scale score decrease.

Four studies including seven experiments analyzed the skindex-10 scale score decrease, The I^2^ test showed I^2^ = 47% < 50%, and Q test showed *P* = 0.08 > 0.05, indicating that slight heterogeneity existed among the studies. The fixed-effects model was used to pool the data, yielding a MD of 4.92 (95% CI 3.47–6.38, Z = 6.63, *P* < 0.00001) (Figure 6).

#### WI-NRS scale score decrease

**FIGURE 7:**
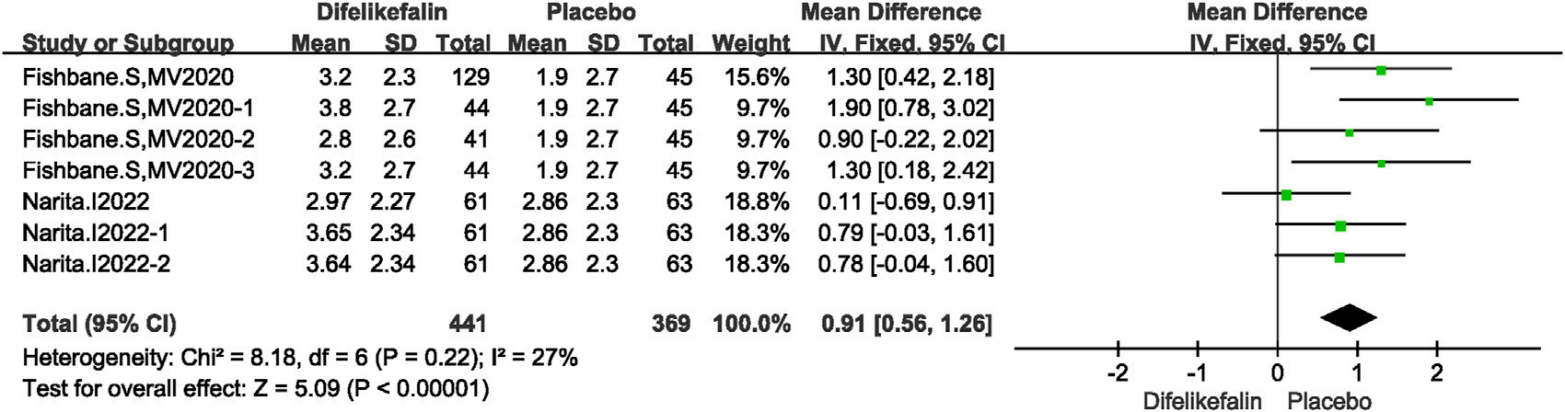
Forest plot showing the WI-NRS scale score decrease.

Two studies including seven experiments analyzed the WI-NRS scale score decrease, The I^2^ test showed I^2^ = 27% < 50%, and Q test showed *P* = 0.22 > 0.05, indicating that slight heterogeneity existed among the studies. The fixed-effects model was used to pool the data, yielding a MD of 0.91 (95% CI 0.56–1.26, Z = 5.09, *P* < 0.00001) (Figure 7).

### Analysis of safety

#### Any TEAE reported

**FIGURE 8:**
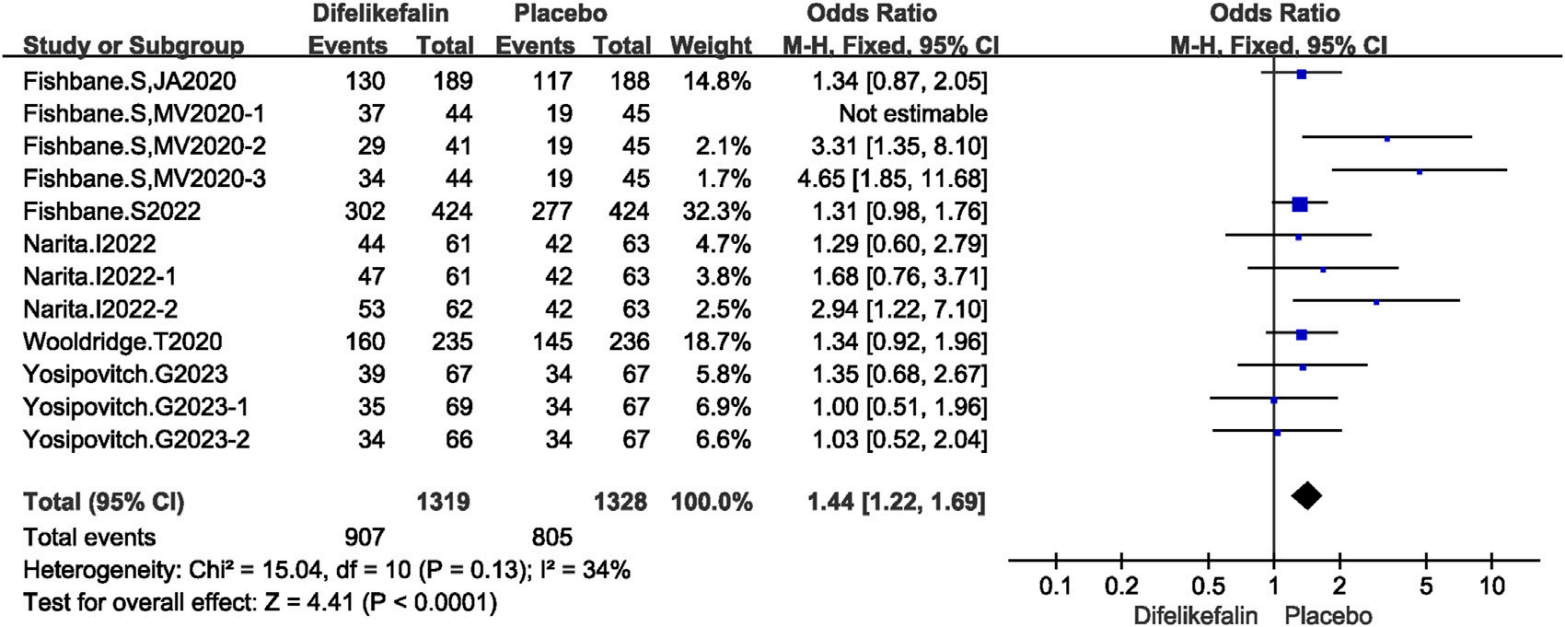
Forest plot showing any TEAE reported.

The incidence of adverse events was greater in the difelikefalin group compared to the placebo group. Six research, including twelve experiments, were examined to determine the occurrence of adverse events. The I^2^ test revealed an I^2^ value of 56%, which is greater than the threshold of 50%, suggesting the presence of heterogeneity among the studies. Additionally, the Q test demonstrated a *P*-value of 0.01, which is less than the significance level of 0.05. After conducting the sensitivity analysis, eleven experiments were included. The I^2^ test showed I^2^ = 34% < 50%, and Q test showed *P* = 0.13 > 0.05, indicating that slight heterogeneity existed among the studies. The fixed-effects model was used to pool the data, yielding an OR of 1.44 (95% CI 1.22–1.69, Z = 4.41, *P* < 0.0001) (Figure 8). Common adverse reactions include diarrhea, dizziness, nausea, headache.

#### Any serious TEAE reported

**FIGURE 9:**
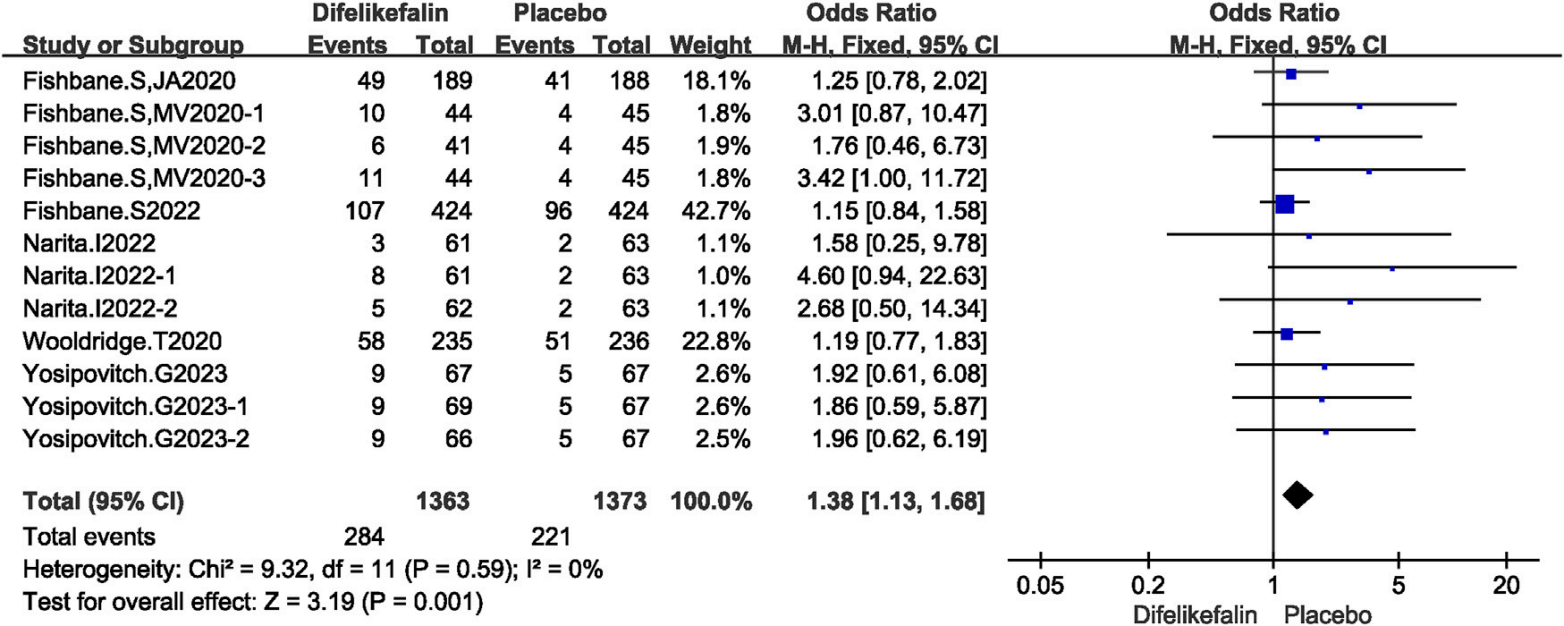
Forest plot showing any serious TEAE reported.

Six studies including twelve experiments analyzed incidence of serious adverse events, The I^2^ test showed I^2^ = 0% < 50%, and Q test showed *P* = 0.59 > 0.05, indicating that no heterogeneity existed among the studies. The fixed-effects model was used to pool the data, yielding an OR of 1.38 (95% CI 1.13–1.68, Z = 3.19, *P* = 0.001) (Figure 9). Incidence of serious adverse events were higher in the difelikefalin group than in the placebo group.

#### Deaths

**FIGURE 10:**
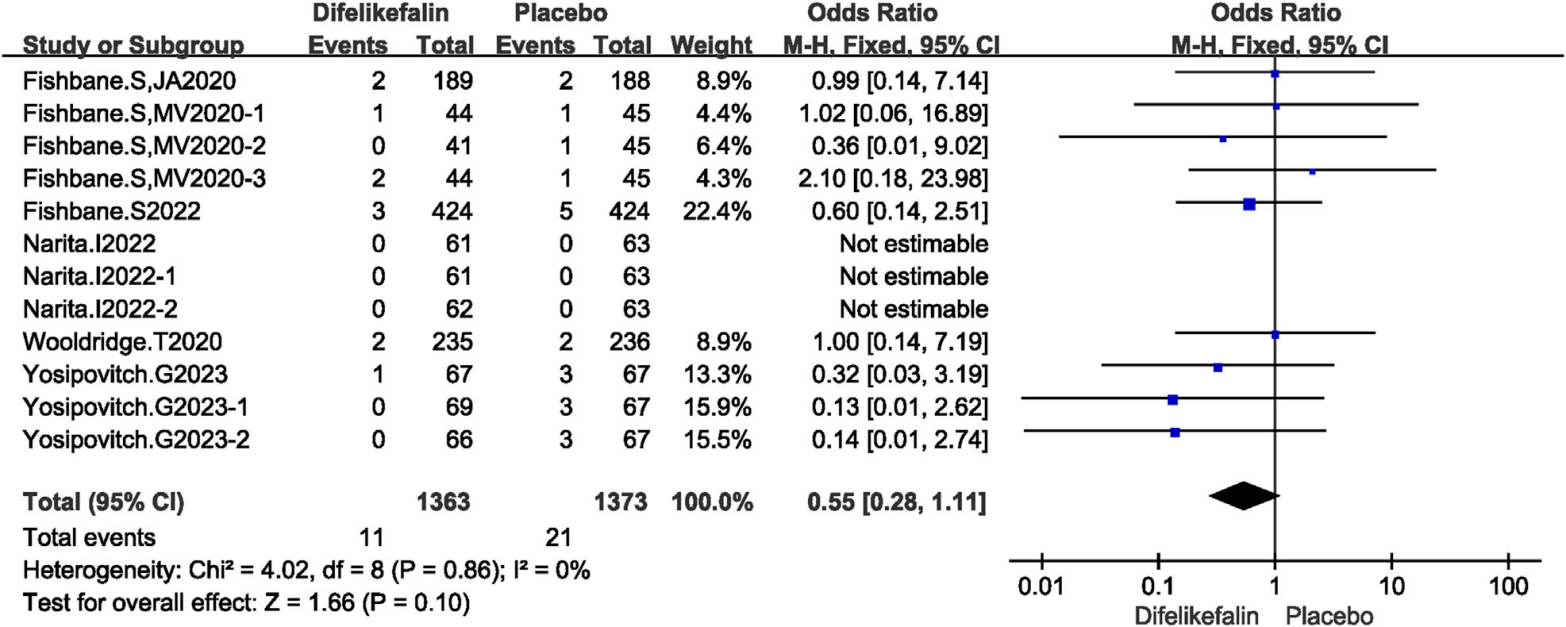
Forest plot showing incidence of deaths.

Six studies including twelve experiments analyzed incidence of deaths, The I^2^ test showed I^2^ = 0% < 50%, and Q test showed *P* = 0.86 > 0.05, indicating that no heterogeneity existed among the studies. The fixed-effects model was used to pool the data, yielding an OR of 0.55 (95% CI 0.28–1.11, Z = 1.66, *P* = 0.10) (Figure 10). There was no significant difference in mortality between the two groups.

#### Bias assessment

**FIGURE 11:**
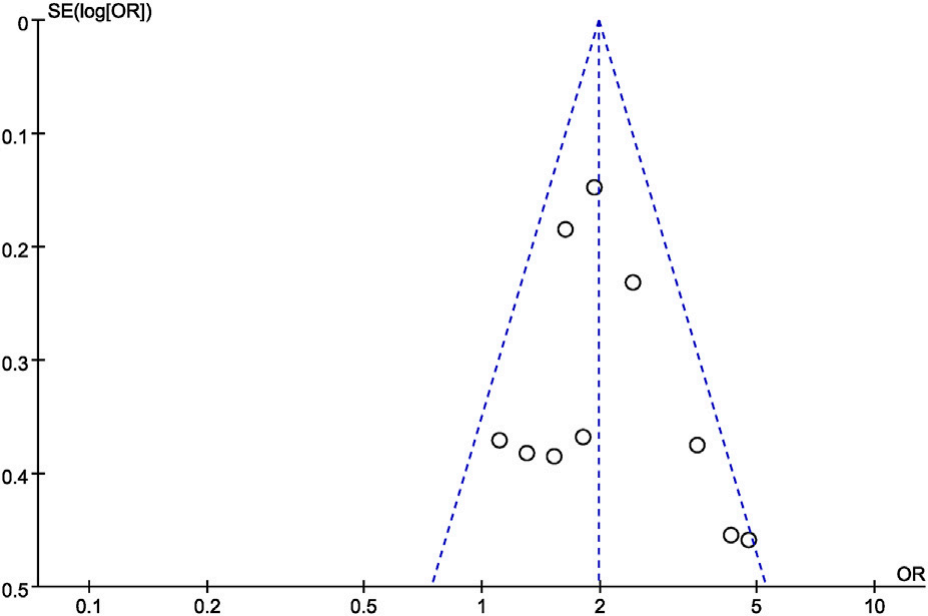
Funnel plot for the score of WI-NRS improvement ≥3-Point from baseline between difelikefalin and placebo group.

Finally, funnel plots were constructed to qualitatively analyze the publication bias among the studies included. The score of WI-NRS improvement ≥3-point from baseline between difelikefalin and placebo group was used as an example. The funnel plots displayed symmetrical distributions, with no obvious publication bias (Figure 11).

## Discussion

The pathogenesis of uremic pruritus has not been completely understood. Many theories have been proposed in numerous studies to explain it. Th1 cells, serum C-reactive protein (CRP), interleukin (IL)-6, and IL-2 levels have been found to be significantly raised in these patients, supporting the significance of inflammation in uremic pruritus (Agarwal et al., 2021). A theory of uremic pruritus pathogenesis implicated toxins in the skin and subcutaneous tissue. Proposed toxins included “uremic toxins,” vitamin A, aluminum, calcium, phosphorus, and magnesium (Verduzco and Shirazian, 2020). One study point that a metabolomic analysis of hemodialysis patients did not identify any solutes associated with pruritus. A role for uremic solutes in pruritus remains to be established (Bolanos et al., 2021). In a study with CKD stage 3–5 managed without dialysis, the authors found that those with moderate to severe pruritus had dry skin as compared to others (Sukul et al., 2019). Yosipovitch et al. (2007) however did not find an association. Szepietowski et al. (2004) have, at least partially, eliminated xerosis as causative of pruritus. Xerosis therefore is more likely to be an exacerbating rather than a causative factor (Makar et al., 2021). One hypothesis implicating an imbalance of opioid system had been proposed, and it emphasized that µ-opioid receptor activation and κ-opioid receptor blockade leading to pruritogenic nerve signaling and pruritogenic cytokines release (Zhang et al., 2023). The lower expression of κ-opioid receptor in uremic pruritus suggests that the peripheral opioid system plays an important role in uremic pruritus (Ko et al., 2023).

The kappa opioid receptor (KOR) is a member of the G-protein-coupled receptor family and its natural endogenous ligand is dynorphin, which decreases synaptic transmission by inhibiting adenylate cyclase and voltage-gated calcium channels and activating voltage-gated potassium channels, resulting in decreased neuronal action potential production and neurotransmitter release (Beck et al., 2019; Zhou et al., 2022). KORs play a critical role in modulating dopamine, serotonin, and glutamate release in the central nervous system. KOR has been implicated in several psychiatric diseases, including schizophrenia, depression, bipolardisorder, and drug addiction (Clark and Abi-Dargham, 2019). Although activation of KOR can inhibit itching, the clinical utility of KOR agonists has been hindered by their dysphoric/psychotomimetic effects, which have been shown to be mediated by activation of central KORs and a downstream beta-arrestin signaling pathway. To avoid producing those adverse effects, novel KOR agonists have been developed through strategies involving G-protein-biased signaling and peripheral restriction. Difelikefalin is a peripheral kappa-opioid receptor agonist that acts primarily on peripheral neurons and cells of the immune system (Fotheringham et al., 2024). Activation of opioid receptors in peripheral neurons reduces afferent impulses to the central nervous system and reduces itching signals. Activating kappa opioid receptors on immune cells, decreases the release of pro-inflammatory chemicals such as IL-6, IL-2 and prostaglandins (Trachtenberg et al., 2020). Difelikefalin is not able to cross the blood-brain barrier due to its small hydrophilic peptide structure. Therefore, unlike many other opioid medications, it does not cause lethal central nervous system side effects such as respiratory depression (Viscusi et al., 2021). Difelikefalin has no action at the mu-opioid receptor, which is responsible for the euphoric effects of traditional opioid medications, so there is negligible abuse potential for this novel agent (Inan and Cowan, 2022). Following a successful phase 3 clinical trial, the FDA has approved the first selective KOR agonist in the US, difelikefalin, which is a peripherally restricted KOR agonist used for treatment-resistant pruritis in patients undergoing hemodialysis.

In recent years, numerous clinical trials have been conducted to evaluate the efficacy and safety of difelikefalin in hemodialysis patients with persistent pruritus. Our study included 4,118 subjects in 9 studies and explored the efficacy and safety of difelikefalin in the treatment of pruritus in hemodialysis patients. To our knowledge, only one relevant meta-analysis has been published, a total of 4 randomized controlled trials were included, it draws a conclusion that difelikefalin can improve itching symptoms in HD patients, it can also increase adverse reactions (Xue et al., 2024), Consistent with our conclusions. But our study had the largest number of included studies and the largest sample size. In this study, a meta-analysis was used to compare the efficacy and safety of difelikefalin and placebo in the treatment of uremic pruritus, providing evidence for clinical use. In the studies, several scales were used to assess the severity of itching, which allowed us to assess the effectiveness of difelikefalin and to compare the results of numerous studies with each other. By comparing the decrease of score of WI-NRS, 5-D itch scale total score and Skindex-10 scale score, difelikefalin can effectively improve the itching symptoms of patients with uremic pruritus. One systematic review shows that difelikefalin, due to its efficacy and good safety profile, can be regarded as the primary treatment for pruritus in patients with chronic kidney disease (Wala and Szepietowski, 2022). Narita I. et al. confirmed that intravenous difelikefalin reduced itching and improved quality of life in patients with moderate to severe pruritus who were undergoing maintenance hemodialysis (Narita et al., 2023). Although difelikefalin can increase adverse reactions, it was well tolerated in participants undergoing HD. Dizziness, Diarrhea, nausea, and headache, which are among the most common TEAEs with difelikefalin. Their incidence was slightly higher than in the placebo group, but not significantly. The risk of death was not statistically different between the two groups. Kraft L. et al. concluded that difelikefalin is effective in the treatment of uremic pruritus, and adverse events were mostly mild in their study population (primarily dizziness, diarrhea and headache) (Kraft et al., 2023). A single-dose, phase 1 study was conducted in healthy subjects and subjects on HD, difelikefalin appeared to have an acceptable safety and tolerability profile with no serious AEs reported. The most common TEAEs were dizziness, headache, paresthesia, and nausea. The majority of TEAEs were reported as mild and considered unrelated treatment (Stark et al., 2023). In a treatment atopic dermatitis’s study, 181 subjects (45.1%) reported 1 or more treatment emergent TEAEs, most were mild or moderate. The most reported TEAEs (>5% of subjects) were abdominal pain/discomfort, nausea, dry mouth, headache, dizziness, and hypertension (Guttman-Yassky et al., 2023).

## Conclusion

Difelikefalin can effectively improve pruritus in patients with uremia. It can also increase adverse reactions; adverse events were mostly mild. The overall quality assessment of the included studies was satisfactory, but some of the included studies were biased by random assignment or blind method. Due to the small sample size of inclusion, further evidence is needed. Indeed, there is no long-term efficacy and safety of difelikefalin. Large, multicenter, high-quality RCTS will be required to provide a basis for clinical drug use.

## Data Availability

The original contributions presented in the study are included in the article/supplementary material, further inquiries can be directed to the corresponding author.
